# Characteristics and outcomes in adult patients with *Staphylococcus lugdunensis* bacteremia compared to patients with *Staphylococcus epidermidis* and *Staphylococcus aureus* bacteremia: a retrospective study in a 16-year period at the university hospital, Japan

**DOI:** 10.1186/s12879-023-08233-9

**Published:** 2023-05-01

**Authors:** Satomi Yukawa, Taro Noguchi, Koh Shinohara, Yasuhiro Tsuchido, Masaki Yamamoto, Yasufumi Matsumura, Miki Nagao

**Affiliations:** 1 Department of Infection Control and Prevention, Kyoto University Hospital, 54 Shogoin Kawahara-Cho, Sakyo-Ku, Kyoto, Postal Code 6068507 Japan; 2 Department of Clinical Laboratory Medicine, Kyoto University Graduate School of Medicine, Kyoto, Japan

**Keywords:** *Staphylococcus lugdunenisis*, *Staphylococci*, Bacteremia, Clinical management

## Abstract

**Background:**

*Staphyococcus lugudnensis* (*S. lugdunensis*) is one of coagulase-negative *Staphylococcus* species with a potential to cause invasive infections. Few studies have evaluated the characteristics and outcomes of patients with *S. lugdunensis* bacteremia (SLB) compared with those of patients with *Staphylococcus epidermidis* (*S. epidermidis*) and *Staphylococcus aureus* (*S. aureus*) bacteremia.

**Methods:**

We performed a single-center retrospective case–control study of patients aged ≥ 18 who had SLB with at least two sets of positive blood cultures at the Kyoto University Hospital, Japan, from January 2005 to June 2022. Patients who had *S. epidermidis* bacteremia (SEB) with at least two sets of positive blood cultures and those who had *S. aureus* bacteremia (SAB) with at least one set of positive blood cultures were randomly selected in a 1:5:5 (SLB:SEB:SAB) ratio.

**Results:**

A total of 22 patients with SLB, 110 patients with SEB, and 110 patients with SAB were included. The proportions of infective endocarditis (IE) and metastatic infections were statistically higher in the SLB group than in the SEB group (14% vs. 2%, *p* < 0.01 and 18% vs. 5%, p 0.02, respectively) and were not significantly different between the SLB and SAB groups (14% vs. 5%, p 0.16 and 18% vs. 16%, p 0.78, respectively). The seven-day mortality was higher in the SLB group than in the SEB group (9% vs. 1%, p 0.02) and similar between the SLB and SAB groups (9% vs. 7%, p 0.77).

**Conclusions:**

The clinical course and outcome of SLB were worse than those of SEB and similar to those of SAB. Appropriate evaluation and treatment for SAB may be warranted in patients with SLB.

## Background

*Staphylococcus lugdunensis* (*S. lugdunensis*) belongs to a group of coagulase-negative *Staphylococci* (CoNS), which was first described by Freney et al. in 1988 [1]. *S. lugdunensis* is a commensal organism present on normal skin in healthy individuals. However, *S. lugdunensis* is also known to have higher pathogenicity than other CoNS. Several virulence factors of *S. lugdunensis* are similar to those of *Staphylococcus aureus* (*S. aureus*) [2]. *S. lugdunensis* causes various infections [3]. Infections attributable to *S. lugdunensis* include bacteremia, infective endocarditis (IE), bone and joint infection, and skin and soft tissue infection (SSTI) [4–7]. Few studies have evaluated the characteristics and clinical outcomes of patients with *S. lugdunensis* bacteremia (SLB) compared with those of patients with *Staphylococcus epidermidis* (*S. epidermidis*) and *S. aureus* bacteremia. *S. aureus* bacteremia (SAB) causes significant morbidity and mortality; complications are frequent, and mortality ranges from 20 to 40% [8]. Mortality for SAB can be improved by clinical management consisting of evidence-based quality-of-care indicators: bundle approach [9]. However, there are no treatment guidelines for SLB due to limited clinical data. To assess appropriate management for SLB, we conducted a retrospective case–control study to investigate the characteristics, clinical courses, and outcomes of patients with SLB compared with those of patients with bacteremia due to *S. epidermidis*, which is the most common pathogen in CoNS-related bacteremia or with SAB [10, 11].

## Methods

### Setting, study design and patients

This retrospective case–control study was conducted at the Kyoto University Hospital, a tertiary care 1,141-bed university hospital located in Japan. From January 1, 2005, to June 30, 2022, patients who had at least one set of blood culture collection were included in this study. Of those, patients with SLB considered clinically significant were included in the analysis. *S. lugdunensis* isolated from two or more consecutive blood cultures of patients was considered clinically significant in the current study. Patients with a single set of positive blood cultures of *S. lugdunensis* were excluded because one set of positive blood cultures for CoNS could have been contaminated. Patients with polymicrobial bacteremia and aged < 18 years were also excluded. Patients who had *S. epidermidis* bacteremia (SEB) with at least two sets of positive blood cultures and those who had SAB with at least one set of positive blood cultures were randomly selected in a 1:5:5 (SLB:SEB:SAB) ratio. This study complied with the Declaration of Helsinki and current ethical guidelines, and it was approved by the research ethics committee at the Kyoto University Hospital (protocol number 3140).

### Definitions and variables

All patients included were reviewed by the infectious disease physician. Chart review was used to collect data. Patient characteristics included age, sex, underlying chronic diseases, the Charlson index of comorbidity, immunosuppressive therapy and chemotherapy within 30 days prior to blood culture collection, the presence of an intravenous catheter and implantable devices, and the category of infection [12]. The illness severity, source of bacteremia, presence of persistent bacteremia, clinical management and outcomes were also reviewed. For the category of infection, each infected case was classified as hospital-acquired, health care-associated, or community-acquired according to the definitions of Friedman et al. [13]. The Pitt bacteremia score was used to evaluate illness severity [14]. Persistent bacteremia was defined as the isolation of *Staphylococcus* in blood cultures obtained for ≥ 3 days despite active antimicrobial therapy according to a susceptibility test [9]. Clinical management included examinations (follow-up blood cultures 48–96 h after antimicrobial therapy was started and echocardiography), early source control (within 72 h from blood culture collection), days to source control, days to appropriate treatment, empiric glycopeptide therapy, early optimal therapy, definitive therapy, combination therapy, and duration of treatment (days). Days to appropriate treatment was defined as the time from blood culture collection to start at least one active drug in accordance with in vitro susceptibility [9, 15]. Empiric glycopeptide therapy was defined as starting glycopeptide drugs within 24 h of blood culture collection. Early optimal therapy was defined as β-lactam antibiotics for methicillin-susceptible isolates and glycopeptide or daptomycin for methicillin-resistant isolates started within 24 h of drug susceptibility being obtained and adjustment of the glycopeptide trough > 15 µg/ml [9]. Definitive therapy was defined as therapy provided after drug susceptibility was obtained. Mandatory intervention began in 2002. Clinical intervention by infectious diseases physician were performed for all patients with bacteremia in our hospital. Infectious disease physicians were immediately informed of a positive blood culture by the laboratory. An infectious disease physician immediately assumed responsibility for a patient with bacteremia and provided recommendations to the attending physician regarding the appropriate approach for the management of bacteremia. Outcomes included the presence of IE, presence of metastatic infections, and mortality. IE was diagnosed according to the modified Duke criteria [16]. Metastatic infection was defined as distant focus that was anatomically unrelated to the primary source.

### Microbiology

Blood cultures were incubated on the BacT/Alert system (bio Mérieux, Marcy l’Etoile, France) for five days. When growth was detected, the sample was subcultured, and an isolated colony was used in the subsequent processes. Identification of clinical isolates was performed as follows: from January 2005 to March 2010, manual techniques; a test for clumping factor (PS LATEX kit, Eiken, Tokyo, Japan) and biochemical properties (ID test SP-18, Nissui Pharmaceutical Co. Ltd, Japan); from March 2010 to December 2016, pos combo 3.1 J panels in the automated MicroScan WalkAway 96 plus system (Siemens, Berlin, Germany); and from January 2017, matrix-assisted laser desorption/ionization time of flight mass spectrometry (MALDI-TOF MS). Antibiotic susceptibilities were determined using the MicroScan WalkAway 96 plus system. Susceptibility of isolates was interpreted according to the Clinical and Laboratory Standards Institute (CLSI) M100-S31 [17]. Oxacillin or cefoxitin susceptibilities were used to detect methicillin resistance according to the CLSI documents. Identification of *S. lugdunensis* isolates prior to January 2017 was reconfirmed by MALDI-TOF MS.

### Statistical analysis

Comparisons among the three groups (SLB, SEB and SAB) were performed using Pearson’s chi-square test for categorical variables and the Kruskal–Wallis test for continuous variables. When a *p* value of < 0.05 was revealed for comparisons among the three groups, comparisons between SLB and SEB and between SLB and SAB were performed using Pearson’s chi-square test or Fisher’s exact test for categorical variables and the Mann–Whitney U test for continuous variables. Kalan-Meier survival analysis and long-rank tests were also used to determine differences in 7-day or 30-day survival among three groups. A *p* value of < 0.05 was considered statistically significant. The statistical analysis was performed using JMP version 16.0 (SAS Institute Inc., Cary, NC, USA).

## Results

### Patient characteristics and illness severity

**Fig. 1:**
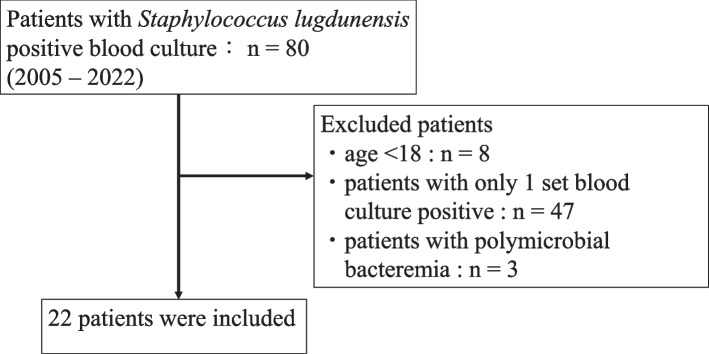
Flow diagram of patients with *Staphylococcus lugdunensis* bacteremia included in the study

There were 80 patients with SLB of at least one set of positive blood cultures, and a total of 22 patients with SLB were included during the study period (Fig. 1). A total of 110 patients with SEB and 110 patients with SAB were also included. Characteristics and outcomes among these three groups are shown in Tables 1 and 2. The proportion of patients with hospital-acquired infection was lower in the SLB group than in the SEB group (45% vs. 92%, *p* < 0.01) and was not significantly different between the SLB and SAB groups (45% vs. 64%, p 0.28). The frequency of methicillin resistance was lower in the SLB group than in the SEB group (23% vs. 83%, *p* < 0.01) and comparable between the SLB and SAB groups (23% vs. 32%, p 0.40). The proportion of hemodialysis in the SLB group was more than twice as high as that in the SAB group (SLB 23%, SAB 11%, p 0.09). The proportion of intravascular catheterization at blood culture collection was lower in the SLB group than in the SEB group (45% vs. 92%, *p* < 0.01) and was not significantly different between the SLB and SAB groups (45% vs. 62%, p 0.15). The proportion of the Pitt bacteremia scores that were four points or more was comparable in each group (SLB 14%, SEB 11%, SAB 9%, p 0.79).

**Table 1:** Characteristics, sources of bacteremia and illness severity of patients with bacteremia

	SLB (*n* = 22)	SEB (*n* = 110)	SAB (*n* = 110)	*P* value among three groups	*P* value SLB—SEB	*P* value SLB—SAB
	N (%)	N (%)	N (%)			
Age (years), median (IQR)	70 (63–80)	64 (50–73)	68 (47–76)	0.09		
Male	11 (50)	68 (62)	59 (54)	0.37		
Healthcare setting				** < 0.01**	** < 0.01**	0.28
Community-acquired	5 (23)	2 (2)	16 (15)			
Health care-associated	7 (32)	7 (6)	24 (22)			
Hospital-acquired	10 (45)	101 (92)	70 (64)			
Methicillin resistance	5 (23)	91 (83)	35 (32)	** < 0.01**	** < 0.01**	0.40
Comorbidities						
Diabetes	7 (32)	22 (20)	20 (18)	0.23		
Hemodialysis	5 (23)	8 (7)	12 (11)	0.09		
Malignancy	13 (59)	66 (60)	50 (45)	0.08		
Solid organ cancer	9 (41)	33 (30)	42 (38)	0.36		
Hematological cancer	4 (18)	37 (34)	9 (8)	** < 0.01**	0.15	0.15
Liver cirrhosis	0	6 (5)	10 (9)	0.24		
Immunosuppressants	7 (32)	53 (48)	31 (28)	** < 0.01**	0.16	0.73
Chemotherapy	6 (27)	45 (41)	22 (20)	** < 0.01**	0.23	0.45
Charlson index, median (IQR)	3 (2–6)	2 (2–4)	3 (2–5)	0.29		
Intravascular catheterization	10 (45)	101 (92)	68 (62)	** < 0.01**	** < 0.01**	0.15
Implantable Devices	5 (23)	16 (15)	28 (25)	0.13		
Intra vascular	2 (9)	6 (5)	11 (10)			
Implantable cardiac	2 (9)	1 1)	6 (5)			
Orthopedic device	1 (5)	8 (7)	13 (12)			
Source of bacteremia						
Intravascular catheter-related	5 (23)	50 (45)	29 (26)	** < 0.01**	**0.048**	0.72
Skin and soft tissue	5 (23)	0	14 (13)	** < 0.01**	** < 0.01**	0.22
Endovascular	5 (23)	5 (5)	13 (12)	**0.02**	** < 0.01**	0.17
Infective endocarditis	3 (14)	2 (2)	6 (5)	**0.04**	** < 0.01**	0.16
Vascular graft infection	0	1 (1)	0	0.55		
Suppurative thrombophlebitis	2 (9)	2 (2)	7 (6)	0.15		
Bone and joint	0	2 (2)	5 (5)	0.34		
Lung	1 (5)	0	6 (5)	**0.01**	0.06	0.9
Intra-abdominal	1 (5)	0	5 (5)	**0.08**		
Other foci	1 (4)	3 (3)	8 (7)			
Unknown	4 (18)	50 (45)	31 (28)	** < 0.01**	0.01	0.11
Eradicable source	14 (64)	56 (51)	58 (53)			
Persistent bacteremia^a^	2/17 (12)	15/93 (16)	22/99 (22)	0.18		
Metastatic infection	4 (18)	5 (5)	17 (16)	**0.01**	**0.02**	0.78
Infective endocarditis or metastatic infection	5 (23)	5 (5)	22 (20)	** < 0.01**	** < 0.01**	0.44
Illness severity						
Pitt bacteremia score, median (IQR)	0 (0, 2)	1 (0, 2)	1 (0, 2)	0.86		
Pitt bacteremia score ≥ 4	3 (14)	12 (11)	10 (9)	0.79		

Data are expressed as numbers (%) unless otherwise indicated

*Abbreviations*: *SLB Staphylococcus lugdunensis* bacteremia, *SAB Staphylococcus aureus* bacteremia, *SEB Staphylococcus epidermidis* bacteremia, *IQR* Interquartile range

^a^The denominator was the number of follow-up blood cultures

**Table 2:** Clinical managements and mortality of patients with bacteremia

	SLB (*n* = 22)	SEB (*n* = 110)	SAB (*n* = 110)	*P* value among three groups	*P* value SLB—SEB	*P* value SLB—SAB
	N (%)	N (%)	N (%)			
Clinical managements						
Follow-up blood cultures	17 (77)	93 (85)	99 (90)	0.21		
Echocardiography	12 (55)	40 (36)	91 (83)	** < 0.01**	**0.11**	** < 0.01**
Early source control	10/14 (71)	43/56 (77)	42/58 (72)	0.84		
Days to source control, median (IQR)	1 (0–2)	1 (0–2)	0 (0–3)	0.35		
Days to appropriate treatment^a^, median (IQR)	0 (0–2)	1 (1–2)	0 (0–1)	** < 0.01**	**0.04**	0.28
Empiric glycopeptide	2 (9)	45 (41)	60 (55)	** < 0.01**	** < 0.01**	** < 0.01**
Early optimal therapy^b^^, c^ (within 24 h)	18/21 (86)	94 (85)	101/106 (95)	**0.046**	0.6	0.1
Definitive therapy^c^						
Cefazolin^d^	9/21 (43)	8 (7)	37/106 (35)	** < 0.01**	** < 0.01**	0.49
Third-generation cephalosporins^e^	2/21 (10)	3 (3)	16/106 (15)	** < 0.01**	0.14	0.50
Cefepime	1/21 (5)	2 (2)	5/106 (5)	0.47		
Oral cephalosporins^f^	2/21 (10)	0	0	** < 0.01**	**0.02**	**0.03**
β-lactam/β-lactamase inhibitors^g^	1/21 (5)	1 (1)	11/106 (10)	** < 0.01**	0.19	0.42
Meropenem	1/21 (5)	1 (1)	5/106 (5)	0.22		
Glycopeptides^h^	5/21 (24)	90 (82)	30/106 (28)	** < 0.01**	** < 0.01**	0.67
Daptomycin	0	5 (5)	2/106 (2)	0.36		
Combination therapy	2 (9)	4 (4)	10 (9)	0.24		
Rifampicin	1/21 (5)	3 (3)	6/106 (6)	0.59		
Gentamycin	1/21 (5)	1 (1)	2/106 (2)	0.47		
Levofloxacin	1/21 (5)	0	0	**0.01**	**0.02**	**0.02**
Clindamycin	0	0	2/106 (2)	0.30		
Duration of treatment (days), median (IQR)	16 (8–27)	13 (10–18)	19 (14–33)	** < 0.01**	0.29	0.20
7-day mortality	2 (9)	1 (1)	8 (7)	**0.04**	**0.02**	0.77
30-day mortality	3/21 (15)	9 (8)	19 (17)	0.13		
Hospital mortality	5/21 (24)	18 (16)	28 (25)	0.24		

Data are expressed as numbers (%) unless otherwise indicated

*Abbreviations*: *SLB* *Staphylococcus lugdunensis* bacteremia, *SAB* *Staphylococcus aureus* bacteremia, *SEB* *Staphylococcus epidermidis* bacteremia, *IQR* Interquartile range

^a^Days to appropriate treatment defined as the time from blood culture collection to the start at least 1 active drug in accordance with in vitro susceptibility

^b^Early optimal therapy defined as starting β lactam antibiotics for methicillin susceptible isolates and glycopeptide or daptomycin for methicillin resistant isolates within 24 h of obtaining drug susceptibility and adjustment of the glycopeptide trough > 15 µg/ml

^c^The denominator was the number of patients who were alive within 24 h of obtaining drug susceptibility

^d^Nafcillin and oxacillin were not available in Japan

^e^Third-generation cephalosporins included ceftriaxone or cefotaxime

^f^Oral cephalosporins included cefalexin or cefcapene pivoxil. Two patients with mild SSTI in the SLB group were treated as outpatients using oral antibiotics

^g^β-lactam/β-lactamase inhibitors included ampicillin/sulbactam or piperacillin/tazobactam

^h^Glycopeptides included vancomycin or teicoplanin

### Source of bacteremia

Intravascular catheter-related bloodstream infection (CRBSI) was the most common source of bacteremia in each group except for unknown focus. CRBSI was less frequent in the SLB group than in the SEB group (23% vs. 45%, p 0.048). The proportion of patients with CRBSI was the same as that of patients with SSTI or with endovascular infection in the SLB group (23%). Cases of SSTI were not observed in the SEB group. The proportion of IE was higher in the SLB group than in the SEB group (14% vs. 2%, *p* < 0.01) and was not significantly different between the SLB and SAB groups (14% vs. 5%, p 0.44). However, the proportion of patients with IE in the SLB group was more than twice as high as that in the SAB group. The proportion of metastatic infections was higher in the SLB group than in the SEB group (18% vs. 5%, p 0.02) and similar between the SLB and SAB groups (18% vs. 16%, p 0.78). All cases with IE or metastatic infections in the SLB group were classified as hospital-acquired or health care-associated (Table 3). The frequency of persistent bacteremia was not significantly different among the three groups (SLB 12%, SEB 16%, SAB 22%, p 0.18).

**Table 3:** Details of patients with *Staphylococcus lugdunensis* bacteremia who had infective endocarditis or metastatic infections

Sex, age (years)	Acquisition	Underling disease(s)	Devices	Methicillin susceptibility	Portal entry	Persistent bacteremia	Detail of infective endocarditis or metastatic infections	Source control^a^	Antibiotics therapy (days)	30-day mortality
Male, 80	Health care- associated	Thyroid cancer, rectal cancer, post AVR	Aortic valve	Susceptible	Unknown	Not persistent	Infective endocarditis (aortic valve) and Spondylitis	Replacement of aortic valve (two days)	Cefazolin (50) and Gentamicin (15) and Rifampicin (43)	Alive
Male, 74	Hospital- acquired	Severe asthma, polymyositis (methylprednisolone 10 mg), SSS	Pacemaker	Susceptible	Unknown	Not persistent	Pacemaker related infective endocarditis (tricuspid valve) and Spondylitis	Eradicate of pacemaker, Tricuspid valve plasty (two days)	Cefazolin (42)	Alive
Female, 84	Health care-associated	Hemodialysis, pancreas cancer, diabetes	Aortic valve	Resistant	Unknown	Persistent	Infective endocarditis (aortic and mitral valves) CT or MRI were not done; thus, metastatic infection was not detected	Not done	Vancomycin (8) and Levofloxacin (7)	Loss of follow up
Female, 69	Hospital- acquired	Aortitis (prednisone 7 mg), cAVB, diabetes	Pacemaker, subclavian artery stent	Susceptible	Unknown	Persistent	Psoas abscess, TEE was not done	Not done	Cefazolin (47)	Alive
Male, 68	Hospital- acquired	Liver cancer, diabetes	Blood accesses catheter	Resistant	Blood accesses catheter	Not persistent	Liver and subcutaneous abscesses, TTE was not done	Not done	Teicoplanin (25)	Alive

*Abbreviations*: *AVR* Atrial valve replacement, *TEE* Transesophageal echocardiography, *TTE* Transthoracic echocardiography, *cAVB* Complete atrioventricular block, *SSS* Sick sinus syndrome, *CT* Computer tomography, *MRI* Magnetic resonance imaging

^a^Source control (Days to source control from blood culture collection)

### Clinical management

Echocardiography was performed less frequently in the SLB group than in the SAB group (55% vs. 83%, *p* < 0.01). Ten patients (45%) in the SLB groups did not receive echocardiography examinations. In detail, echocardiography was not recommended by Infectious disease physicians for six patients. Echocardiography was recommended for three patients, however, attending physicians did not follow the recommendations of infectious disease physicians. One patient died until isolates from blood culture were identified as *S. lugdunensis*, therefore, an infectious disease physician could not intervene enough. There were no significant differences in the proportion of follow-up blood cultures, early source control, early optimal therapy, and combination therapy between the SLB and the other groups. The number of days between the start of appropriate therapy and the blood culture collection was lower in the SLB group than in the SEB group (median [IQR]: SLB, 0 [0–2] vs. SEB, 1 [1-2], p 0.04) and similar between the SLB and SAB groups (median [IQR]: SLB, 0 [0–2] vs. SAB, 0 [0–1], p 0.28). Empiric glycopeptide therapy was prescribed less frequently in the SLB group than in the SAB group (9% vs. 55%, *p* < 0.01) and in the SEB group (9% vs. 41%, *p* < 0.01). As definitive therapy, Cefazolin was prescribed more frequently in the SLB group than in the SEB group (43% vs. 7%, *p* < 0.01). Glycopeptide therapy was prescribed less frequently in the SLB group than in the SEB group (24% vs. 82%, *p* < 0.01). The duration of treatment (days) in the SLB group was comparable to that in the other groups (median [IQR]: SLB, 16 [8-27] vs. SEB, 13 [10-18], p 0.29, and median [IQR]: SLB, 16 [8-27] vs. SAB, 19 [14-33], p 0.20, respectively).

### Outcomes

**Fig. 2:**
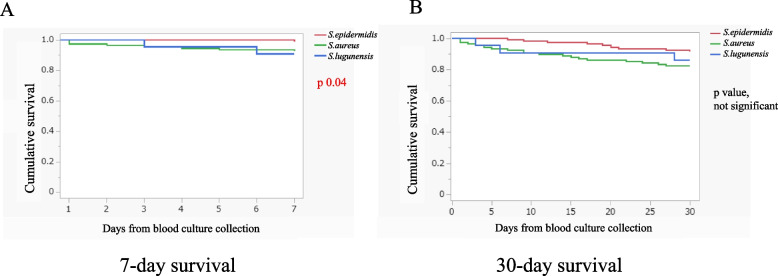
Survival curves of patients with bacteremia caused by different *Staphylococcus* species. **A** 7-day survival from blood culture collection (Long-rank test: p 0.04). **B** 30-day survival from blood culture collection (Long-rank test did not reveal significant difference among three groups)

The seven-day mortality was higher in the SLB group than in the SEB group (9% vs. 1%, p 0.02) and similar between the SLB and SAB groups (9% vs. 7%, p 0.77) (Table 2 and Fig. 2A). Patients with SLB who died within seven days of blood culture collection were classified as health care-associated due to the requirement for chronic hemodialysis (Table 4). There were no differences in 30-day and hospital mortalities among the three groups (SLB 15%, SEB 8%, SAB 17%, p 0.13 and SLB 24%, SEB 16%, SAB 25%, p 0.24, respectively) (Table 2 and Fig. 2B). The thirty-day mortality in the SLB group was approximately twice as high as that in the SEB group.

**Table 4:** Details of patients with *Staphylococcus lugdunensis* bacteremia who had died within seven days

Sex, age (years)	Acquisition	Underling disease(s)	Devices	Methicillin susceptibility	Portal entry	Persistent bacteremia	Detail of Metastatic infections	Source control ^a^	Antibiotics therapy (days)	Days to die from blood culture collection
Male, 63	Health care- associated	Hemodialysis, hepatocellular carcinoma,	No	Susceptible	Unknown	Follow-up blood culture was not done	Lung abscess (suspicion of septic emboli), TTE was not done	Not done	Tazobactam/piperacillin (3) and vancomycin (1)	Three days
Female, 87	Health care- associated	Hemodialysis, diabetes, cAVB	Peripheral vein catheter	Susceptible	Unknown	Follow-up blood culture was not done	Not investigated, TTE was not done	Not done	Tazobactam/piperacillin (5), meropenem (1) and vancomycin (3)	Six days

*Abbreviations*: *TTE* Transthoracic echocardiography, *cAVB* Complete atrioventricular block

^a^Source control (Days to source control from blood culture collection)

## Discussion

In the current study, we demonstrated that the proportions of IE, metastatic infections and seven-day mortality in the SLB group were higher than those in the SEB group and similar to those in the SAB group.

The thirty-day mortality in the SLB group was approximately twice as high as that in the SEB group in our study. Other reports revealed that 30-day mortality was 11–14.3% in patients with SLB [18, 19]. Lin et al. reported that hospital mortality was 20.8% in a study consisting of 41 cases with SLB and seven cases of sterile site infection with *S. lugdunensis* [15]. The thirty-day and hospital mortalities in the SLB group in our study were consistent with those in previous reports. Therefore, SLB has been associated with high mortality. The proportion of IE in the SLB group was more than twice as high as that in the SAB group. Previous reports have shown that the proportion of IE by *S. lugdunensis* was is 8–27% [15, 19, 20]. Our results were also consistent with previous reports. Two of the four patients with metastatic infections in the SLB group had multiple deep abscesses (psoas abscess and liver and subcutaneous abscesses). Several case reports described multiple deep abscesses and a metastatic infection following an abscess caused by *S. lugdunensis* as well as our cases*.* Previous reports revealed that psoas abscesses are attributable to *S. lugdunensis* [21, 22]. Another report showed that a patient who had IE following a gluteal abscess was infected with *S. lugdunensis* [23]. The proportion of metastatic infections in the SLB group was almost the same as that in the SAB group. Thus, SLB frequently causes complicated infections.

The proportions of IE, metastatic infections and seven-day mortality were higher in the SLB group than in the SEB group, despite the higher proportions of methicillin resistance and the delay to start appropriate treatment in the SEB group. These results suggest the high pathogenicity of *S. lugdunensis* compared with other CoNS, such as *S. epidermidis*. *S. lugdunensis* has several virulence factors, including synergistic hemolytic peptides, von Willebrand factor-binding protein, fibrinogen-binding protein, Lugdulysin (metalloprotease), iron-regulated surface determinant (Isd) proteins, nuclease, and IsdC, which are associated with biofilm formation [2, 24, 25]. These virulence factors give *S. lugdunensis* the potential to cause aggressive infections.

The clinical management of SLB needs to be improved because *S. lugdunensis* has high pathogenicity, and SLB is associated with complicated infections and high mortality. A recent study revealed that patients with IE caused by *S. lugdunensis* died significantly earlier than those with IE caused by *S. aureus* or other CoNS [26]. Another study reported that medical treatment alone was an independent risk factor for the mortality of IE caused by *S. lugdunensis* [27]. Additionally, IE caused by *S. lugdunensis* is associated with high mortality [28, 29]. Thus, prompt and appropriate clinical management is required. Echocardiography was performed for only half of the patients with SLB. IE caused by *S. lugdunensis* might be underestimated because the proportion of patients that undergo echocardiography was lower among patients with SLB than among those with SAB. Another study demonstrated that for patients who had SLB with at least two sets of positive blood cultures, 25% of patients had IE; thus, the growth of *S. lugdunensis* in two separate blood cultures should prompt the consideration of work-up for IE [19]. A recent study reported that patients with SLB that had a bedside infectious disease specialist consultation had transthoracic echocardiography performed more often as well as a lower 90-day and one-year mortality [30]. Echocardiography is recommended for patients with SAB, but it should also be performed for patients with SLB. The proportion of patients that received echocardiography examinations was low in the SLB group despite intervention by infectious disease physicians in our study. This study included several cases in old period. Invasiveness of *S. lugdunensis* or importance of echocardiography for SLB were not recognized before. Further, although intervention was mandatory, attending physicians were not required to follow the recommendations of the infectious disease physicians. An approach that allows echocardiography to be performed is warranted. Zinkernagel et al. found that IE caused by *S. lugdunensis* is a community-acquired infection, and IE occurred far less frequently in a nosocomial setting [31]. However, our patients with IE in the SLB group were not classified as community-acquired cases. This may be because of differences in the patient population. Patients with SLB should be carefully examined for signs of IE even in health care-associated or hospital-acquired settings. Furthermore, we demonstrated that the clinical outcome for patients with SLB was similar to that for patients with SAB. Appropriate evaluation and treatment for patients with SAB may also be warranted for patients with SLB.

The proportion of patients requiring hemodialysis in the SLB group was more than twice as high as that in the SAB group. A recent report found that patients who had SLB with two sets of positive blood cultures were more likely to be on hemodialysis than those with one set of positive blood cultures [32]. It is unclear whether an altered cutaneous microbiological flora in end-stage renal disease patients can influence this result; hemodialysis may lead to the acquisition of SLB [33]. Both patients with SLB who died within seven days of the blood culture collection were on chronic hemodialysis. Previous studies have shown that SAB in patients on hemodialysis is frequently associated with high mortality [34, 35]. This may be applicable to patients with SLB.

The proportion of methicillin resistance was 23% in the SLB group. In general, methicillin resistance is rare in *S. lugdunensis* [36, 37]. However, recent reports have demonstrated high proportions of methicillin resistance in *S. lugdunensis*. The frequencies of methicillin resistance were 33.3% in Taiwan and more than 40% in Iraq, respectively [38, 39]. These reports and our result may indicate the regional spread of *S. lugdunensis* with methicillin resistance in the parts of the world. The caution is needed for methicillin resistance of *S. lugdunensis*, and a multicenter study is required for confirming whether there is a trend toward increasing the proportion of methicillin resistance for *S. lugdunensis* in Japan.

The current study had some limitations. First, it was a retrospective and single-center study. Therefore, the possibility of unintentional selection bias in the selection of patients cannot be fully excluded. Furthermore, because our hospital is a university hospital, there might have been intentional treatment and possible hospital bias. Second, our study had a small number of patients with SLB. This study was underpowered to detect any small differences between the groups, and statistically significant differences should be interpreted with caution. Further studies are warranted to confirm our findings. Even so, to our knowledge, this study included the largest SLB cohort for comparison with SAB and SEB cohorts.

## Conclusions

In conclusion, we demonstrated that the proportions of IE, metastatic infections and seven-day mortality in patients with SLB were higher than those in patients with SEB and similar to those in patients with SAB**.** The proportion of echocardiography examinations was low in the SLB group. Appropriate evaluation and treatment recommended for patients with SAB may also be warranted for patients with SLB.

## Data Availability

The datasets generated and/or analyzed during the current study are available from the corresponding author on reasonable request.

## Abbreviations

*S. lugdunensis*: *Staphyococcus lugudnensis*
*S. aureus*: *Staphylococcus aureus*
*S. epidermidis*: *Staphylococcus epidermidis*
SLB: *Staphyococcus lugudnensis* Bacteremia
SEB: *Staphylococcus epidermidis* Bacteremia
SAB: *Staphylococcus aureus* Bacteremia
IE: Infective endocarditis
CoNS: Coagulase-negative *Staphylococci*
SSTI: Skin and soft tissue infection
MALDI-TOF MS: Matrix-assisted laser desorption/ionization time of flight mass spectrometry
CLSI: Clinical and Laboratory Standards Institute
CRBSI: Catheter-related bloodstream infection
IQR: Interquartile range
Isd: Iron-regulated surface determinant
